# Comparative and Systematic Omics Revealed Low Cd Accumulation of Potato *StMTP9* in Yeast: Suggesting a New Mechanism for Heavy Metal Detoxification

**DOI:** 10.3390/ijms221910478

**Published:** 2021-09-28

**Authors:** Dandan Li, Guandi He, Weijun Tian, Muhammad Saleem, Yun Huang, Lulu Meng, Danxia Wu, Tengbing He

**Affiliations:** 1 College of Agricultural, Guizhou University, Guiyang 550025, China; gs.lidd19@gzu.edu.cn (D.L.); gdhe@gzu.edu.cn (G.H.); gs.tianwj18@gzu.edu.cn (W.T.); gs.yunhuang19@gzu.edu.cn (Y.H.); gs.llmeng18@gzu.edu.cn (L.M.); gs.wudx19@gzu.edu.cn (D.W.); 2 Department of Biological Sciences, Alabama State University, Montgomery, AL 36104, USA; msaleem@alasu.edu; 3 Institute of New Rural Development, Guizhou University, Guiyang 550025, China

**Keywords:** metal tolerance proteins, specific evolutionary analysis, cadmium tolerance, *StMTP9*

## Abstract

The metal tolerance protein (MTP) family is a very old family with evolutionary conservation and less specific amplification. It seems to retain the original functions of the ancestral genes and plays an important role in maintaining metal homeostasis in plant cells. We identified the potato *MTP* family members for the first time, the specific and conservative *StMPT*s were discovered by using systematic and comparative omics. To be surprised, members of the *StMTP* family seem to have mutated before the evolution of dicotyledon and monocotyledon, and even the loss of the entire subfamily (subfamily G6, G7). Interestingly, *StMTP9* represents the conserved structure of the entire subfamily involved in toxic metal regulation. However, the gene structure and transmembrane domain of *StMTP8* have undergone specific evolution, showing that the transmembrane domain (Motif13) located at the NH_2_ terminal has been replaced by the signal peptide domain, so it was selected as the control gene of *StMTP9*. Through real-time fluorescence quantitative analysis of *StMTP*s under Cd and Zn stress, a co-expression network was constructed, and it was found that *StMTP9* responded significantly to Cd stress, while *StMTP8* did the opposite. What excites us is that by introducing *StMTPs 8/9* into the *∆ycf1* yeast cadmium-sensitive mutant strain, the functional complementation experiment proved that *StMTPs 8/9* can restore Cd tolerance. In particular, *StMTP9* can greatly reduce the cadmium content in yeast cells, while *StMTP8* cannot. These findings provide a reference for further research on the molecular mechanism of potato toxic metal accumulation.

## 1. Introduction

Heavy metals are non-essential element for plant growth and development. They can cause damage to plants even at low concentrations [1,2] and easily absorbed by plant roots through Zn^2+^, Fe^2+^ and Ca^2+^ transporters [3]. Their accumulation in plants can seriously affect crop yields, varieties, and even endanger human health [4,5]. Therefore, it is urgent to reduce the entry of heavy metals into crops and reduce the risk of animals and humans being poisoned by them. It has been reported that some plants have special molecular mechanisms in terms of the accumulation mechanism of heavy metals [6], but the mechanism of cadmium accumulation in potato is still unknown, and the specific functional genes and proteins remain to be discovered.

The heavy metal Cd is a metal element with very strong migration ability, and its migration conversion rate is several times or even dozens of times that of other metal elements. According to research reports, it seems that specialized Zn^2+^ channels can often also significantly absorb Cd^2+^ [7]. This makes us interested in co-expressed genes under zinc and cadmium exposure, which may help us find special potato genes that use Zn^2+^ channels to transport Cd^2+^. Many plants are poisoned by heavy metals, so they must respond quickly to minimize metabolic loss [8]. Plants have gradually evolved specific mechanisms such as transmembrane transport to actively absorb and discharge these metals, thereby regulating their concentration in cells [9]. It is the transmembrane transpot that affects the absorption, translocation and distribution of metal ions in plant cells and tissues. The transport proteins in plants mainly include ATP-Bing Cassette (ABC) [10], heavy metal ATPase (HMA) [11], natural resistance-associated macrophage protein (Nramp) [12] and cation diffusion facilitator (CDF), which is also called MTP.

The CDF which composed by membrane-bound proteins can maintain the cell homeostasis of metal ions in plants [13]. They have a central role in the tolerance to heavy metal stress of plants [14]. Their function is to regulate metal homeostasis and can transport divalent cations commonly found in archaea, bacteria and eukaryotes [15]. In plants, CDF transporters are called MTP because they are responsible for transporting and separating metals into vacuoles. It is reported that in addition to transporting metal ions, MTP is also involved in processes such as signal transduction, anti-oxidative stress and plant nodule development [16,17,18]. Because of the different substrate characteristics, the MTP family can be divided into three sub-families: Mn-MTP, Zn-MTP and Zn/Fe-MTP. The carboxyl cytoplasmic end and 4-6 transmembrane domains (TMD) are usually found in these members [19]. Highly conserved characteristic sequences exist in TMD2 and TMD5 [20]. *Arabidopsis thaliana MTP1* (*AtMTP1*) is the first MTP protein identified in plants [21]. overexpressing *AtMTP1* in Arabidopsis has a strong resistance to high Zn^2+^ stress, and it significantly increase the Zn^2+^ concentrations in root [22]. Many studies have been reported that MTP protein plays an important role in different plants, and it can transport various divalent ions [23,24]. For example, under the stress of excessive Zn^2+^ and Cd^2+^, the expression of *MTP1*, *MTP3* and *MTP4* in *Citrus sinensis* significantly up-regulated in roots or leaves [25]. In tobacco, overexpression of *Oryza sativa MTP1* (*OsMTP1*) can reduce the levels of phytotoxicity caused by Cd^2+^ stress [26]. The *Triticum urartu MTP1* can maintain ion balance in plants by isolating excess Zn^2+^ and Co^2+^ in vacuoles [27]. The heterologous expression of tea *MTP8.2* in Arabidopsis gives it tolerance to Mn^2+^ [28]. In yeast cells expressing *Populus trichocarpa MTP8.1*, *MTP9* and *MTP10.4* can transport Mn^2+^, while *MTP6* is related to the Co^2+^, Fe^2+^ and Mn^2+^ [29]. It also indicated that the *OsMTP11* is a trans-Golgi transporter of Mn^2+^ [30]. These all show the important role of MTP gene (MTP) in response to heavy metal stress. Up to now, 12 *MTPs* have been identified in Arabidopsis [28]. 13 *MTPs* were found in *Camellia sinensis* [22], and 11 members were found in *Vitis vinifera* [31]. The MTP in potato have rarely been reported in. However, the Yunshu 505, a low-cadmium-enriched potato variety, is expected to provide clues for finding genes for low-cadmium accumulation.

On the basis of our previous studies, it was found that cadmium enrichment ability of different potato varieties varied greatly. Therefore, in this study, specific or representative genes in *StMTP*s were obtained through whole-genome, systematics and qRT-PCR analysis. The core StMTPs with different accumulative amounts and tolerance were identified by comparative omics and cell biology methods. These results provide important reference for molecular breeding of potato varieties with low Cd accumulation, and also provide bidirectional selection for soil remediation and environmental management of potato varieties with high Cd accumulation.

## 2. Results

### 2.1. Chromosome Locations and Collinearity Analysis

**Figure 1:**
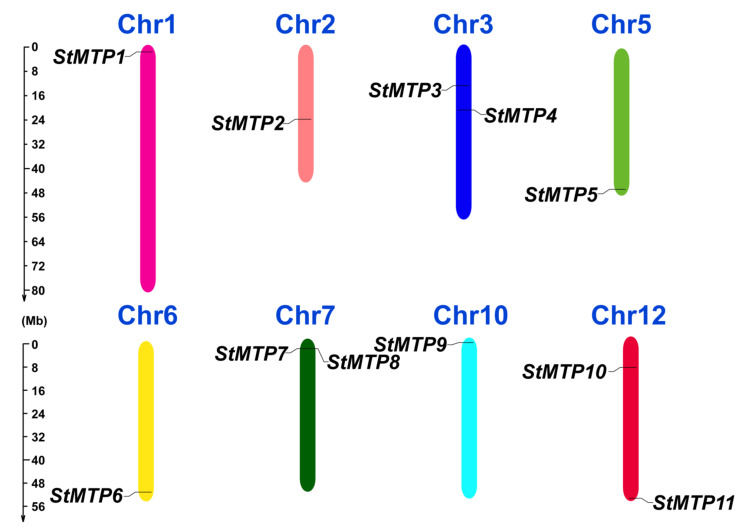
Distributions of *StMTP*s on the chromosomes of *Solanum tuberosum*. Different chromosomes are marked with different colors, and the number of them are indicated at the top of each chromosome. The length of the chromosome is indicated by the scale ruler on the left.

Eleven *StMTP*s were identified in the potato genome files and named according to their position on the chromosome (Table S1). It can be seen from Figure 1 that these 11 genes are mainly distributed on eight chromosomes. Among them, except for Chr3 and Chr7, two genes are distributed, and the rest of the chromosomes contain only one StMTP. What has caught our attention is that StMTP7 and StMTP8 are located very close to each other, but there is no tandem duplication of these two genes.

**Figure 2:**
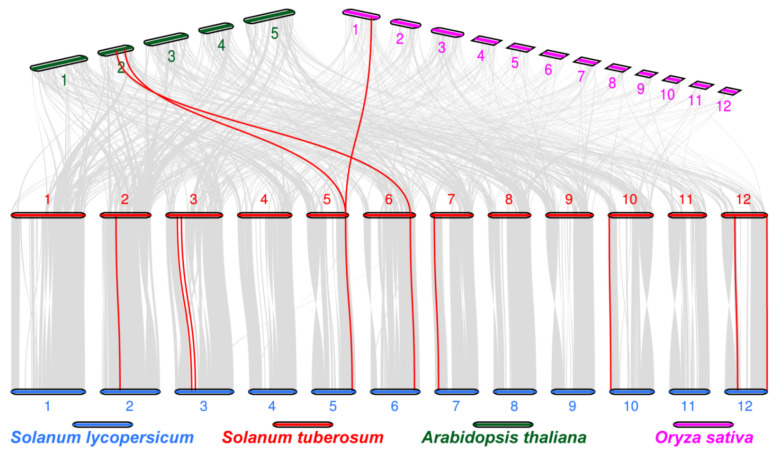
Collinearity analysis of the MTPs in *Arabidopsis thaliana, Oryza sativa, Solanum lycopersicum*, and *Solanum tuberosum*. Different colors represent chromosomes of different species, and the number of each chromosome is labeled at their top or bottom. The gray lines indicate the collinear block of genome of the plants and the red line indicates the synonymous MTP gene pairs in potato.

In order to further study the evolutionary mechanism of MTP in potatoes, we selected tomato, Arabidopsis and rice for collinearity analysis. 12 collinearity gene pairs were found in the 4 species. The most collinearity gene pairs (nine pairs) were found in potato and tomato (Figure 2), both of which belong to Solanaceae, indicating that they have a high degree of genetic similarity. In potato, there is a special gene (*StMTP5*) has a continuous collinearity gene pair in tomato, Arabidopsis and rice. What surprised us is that StMTP1 and StMTP8 have no collinearity gene pair, even in tomato.

### 2.2. Phylogeny Analysis and Classification of Potato MTPs

**Figure 3:**
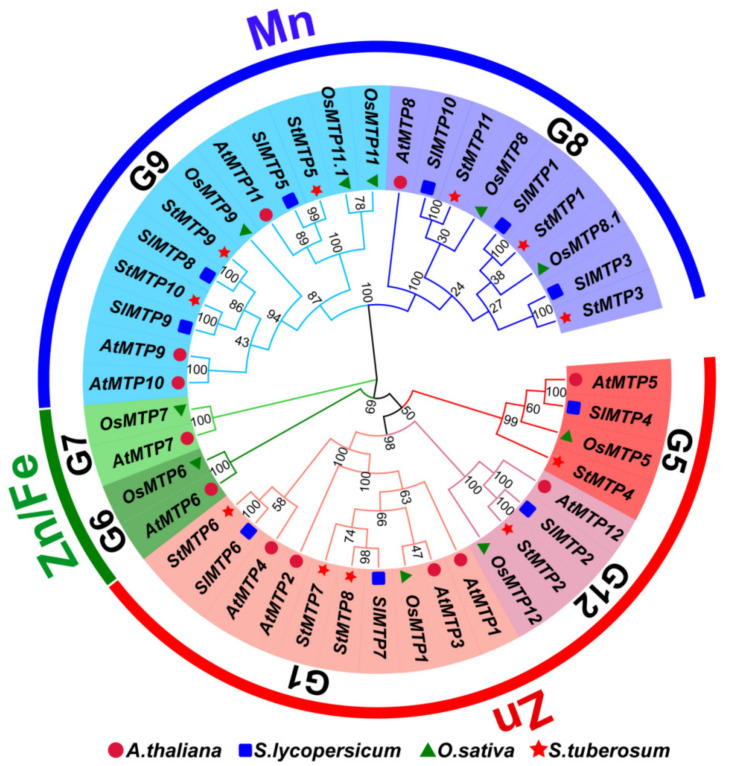
The Neighbor Join (N-J) method in MEGA-X (v10.1.8) software is used to construct a phylogenetic tree. Red five-pointed stars are used to indicated as potatoes, tomatoes were expressed as blue squares, red solid circles are used to represent Arabidopsis, and green triangles are used to represent rice.

The MTP protein sequences of Arabidopsis (12), rice (10), tomato (10) and potato (11) were used to construct a phylogenetic tree (Figure 3) to explore the evolution of StMTP and its subfamily classification. The MTP family is mainly divided into three sub-families (Mn-MTP, Zn/Fe-MTP, Zn-MTP). And they can be further subdivided into seven different groups (G5, G12, G1, G6, G7, G9, G8). What is interesting is that we did not discover a member of the Zn/Fe-MTP subfamily (G6 and G7). The AtMTP12 and StMTP2 are in adjacent branches, while AtMTP5 and StMTP4 are closer. The StMTP7 very close to StMTP8, indicating that their protein sequences are similar.

### 2.3. Gene Structure and Motif Analysis

**Figure 4:**
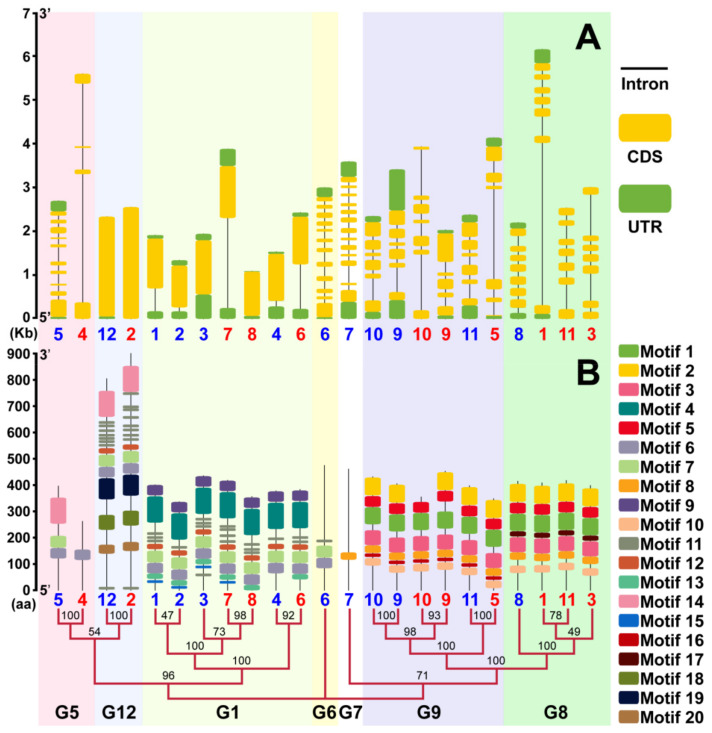
StMTPs structure analysis and conservative motif analysis. StMTPs 1–11 is marked as red 1–11, blue numbers 1–12 represent AtMTP1-AtMTP12. (**A**): The exon-intron structure of *StMTP*s, the black line represents the introns, the yellow box denotes the untranslated 5′ and 3′ regions of the UTR and the green box indicated as the exons. The length of the exons, introns and untranslated regions were estimate by the scale bar. (**B**): Schematic diagram of conserved motifs. The different themes were represented by different colored boxes and the length of each sequence were marked. The scale bar on the left indicates the length of the MTP protein sequence.

In the structure of genes, the exons and introns play an essential role in the analysis of gene family evolution [32]. None of the genes in G12 have introns, the genes in G1 have only one intron at most, the remaining groups contain 3–12 introns (Figure 4A). In G1, StMTP8 does not contain introns, and the UTR at the 5’ end is shorter. Similarly, compared with other genes in G9, the StMTP9 contains shorter introns than others.

Twenty motifs have been found in Arabidopsis and potato members of the MTP gene family. The number and type of motifs are basically the same in the same group. However, StMTP4 contains only one motif6, which is two motifs less than AtMTP5. several unique motifs were contained in each group. Such as the motif 19 that was only found in group 2, and the motifs 4, 12 and 9 that were only found in group 3.

### 2.4. The Features of StMTP Proteins

The characteristics of StMTP proteins are predicted by an online software, and the results are shown in Table 1. The length of these proteins are 28.77–100.11 KDa, the isoelectric points varied from 5.01 to 8.96. The stability of a protein is judged by the protein instability index, and its value >40 indicates an unstable protein. It can be seen from the table that 27% of the proteins are stable, and the remaining 73% are unstable. The interesting thing is that eight proteins in potato were hydrophobic, which also account for 73% of the total proteins. The results of subcellular localization showed that all of them were located in vacuoles. However, StMTP10 and StMTP9 were also present in the cell membrane. All StMTPs contain 2–16 TMDs. Compared with other proteins, StMTP2 contains the most TMD (16), and StMTP4 has the least TMD (2).

### 2.5. The Characteristics of Amino Acid Sequence in StMTPs Subfamily

**Figure 5:**
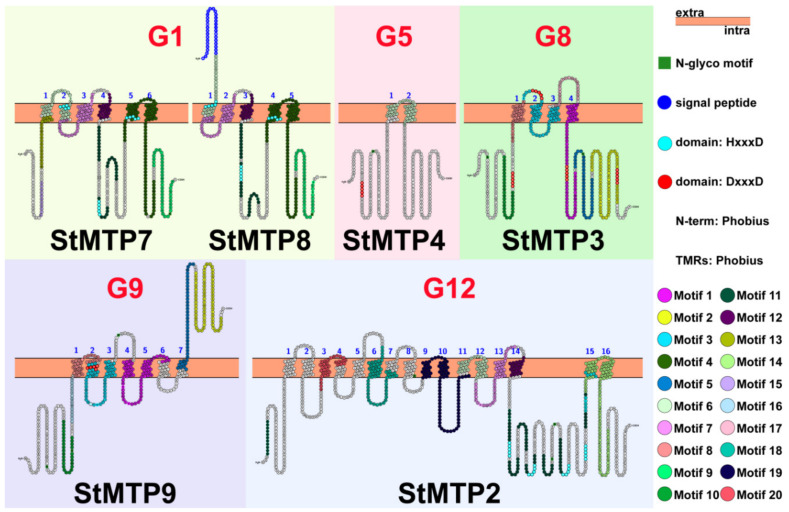
StMTPs protein sequence analysis. The orange box represents the biofilm. Different motifs are represented by circles (black fonts) of corresponding colors, The blue number indicates the number of the transmembrane domain. HXXXD residues are represented by sky blue circles (white font), and DXXXD residues are marked by dark red circles (white font). The dark blue circle with white font indicates the presence of a single peptide in the gene. The blue number stands for the transmembrane domain.

We can discover that the protein sequences of StMTP were similar to that of rice and Arabidopsis. In addition, the amino acid sequences of potato and Arabidopsis in the Mn-MTP subfamily are more similar than those of Zn-MTP, which indicates that genes in the subfamily are more highly conserved. The aspartic (D) DXXXD (X = any amino acid) residues were found in the Mn-MTP. HXXXD residues containing aspartic and histidine (H) were found in Zn-MTP (Figure S1). A large number of studies have reported that HXXXD in Zn-MTP and DXXXD in Mn-MTP are both on TMD2 and/or TMD5 [33,34]. Histidine-rich regions are also found in the Zn-MTP subfamily. According to the results of phylogeny, gene structure and motif analysis, 1–2 representative genes were selected from each group and conduct in-depth exploration of their protein sequences. But we were surprised to find that only the conserved domains of StMTP7 (HXXXD) and StMTP9 (DXXXD) are located on TMD2 among the six genes. It can also be seen in the figure that each gene contains an N-glyco motif. In particular, a signal peptide was found on StMTP8 (Figure 5).

### 2.6. The Cis-Acting Elements in the Promoters of StMTPs

**Figure 6:**
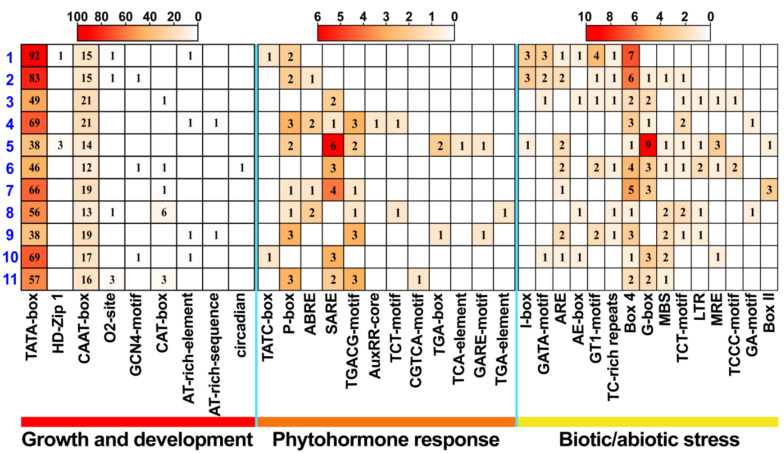
The promoter sequences of the 2000 bp upstream region of the start codon of the *StMTP*s were analyzed using Plantcare. The abscissa represented the type of promoter, and the ordinate represents the specific gene. The blue numbers 1–11 indicate *StMTPs 1–11*. All promoters can be divided into three categories: growth and development, plant hormone response, and biotic/abiotic stress-related.

The promoter sequences of the 2000 bp upstream region of the start codon in the *StMTPs 1–11* were obtained and analyzed by Plantcare. All *cis*-acting elements can be divided into growth and development, phytohormone response, and biotic/abiotic stress three major categories. Our results show that all *StMTP*s contain a mass of promoter core elements such as the CAAT- and the TATA-boxes (Figure 6). Many light -responsive elements such as GATA-motif, Box-4, AE-box, G-box and MRE were contained by each of them. There are some *cis*-acting elements that respond to hormones (auxin, salicylic acid, abscisic acid and methyl jasmonate) include the AuxRR-core, SARE, the TCA-element, and the ABRE and the CGTCA-motif. Also, some uncommon functional elements were found such as the CAT-box that relates to meristem expression, the *cis*-acting regulatory element involved in zein metabolism regulation (O2-site). HD-Zip1 is related to the differentiation of palisade mesophyll cells, and the RY-element is involved in seed-specific regulation (Table S6).

### 2.7. The Response of StMTPs in Different Tissues to Heavy Metal Stress

**Figure 7:**
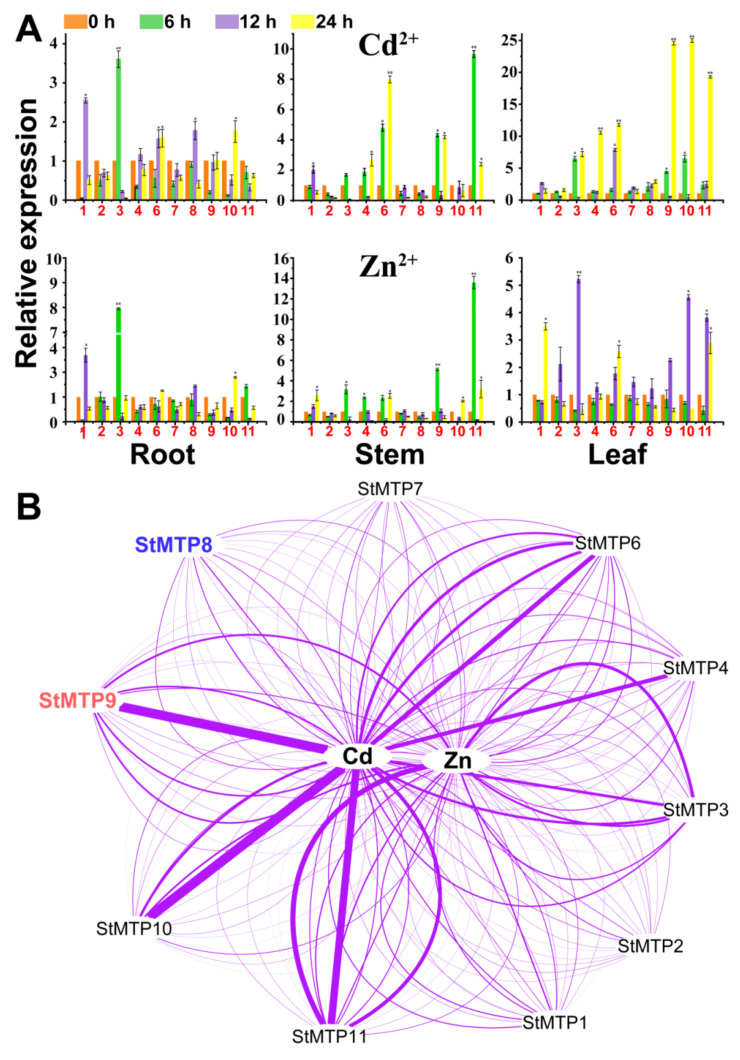
The expression analysis of *StMTP*s under Zn and Cd stress. (**A**): The expression profile of StMTPs in root, steam and leaf. The *StMTPs* *1–11* were abbreviated as red font 1–11. * = *p* ≤ 0.05, ** = *p* ≤ 0.01. (**B**): A visual analysis network for the influence of heavy metals on *StMTP*s expression. The wider connection lines between the metal and the gene, the higher expressions of the gene under the metal stress conditions. *StMTP8* marked in blue and *StMTP9* were marked in red font were used for subsequent heterologous expression in yeast.

Real-time quantitative PCR was used to detect the expression of *StMTP*s under the heavy metal Zn and Cd stress. The expression at different times and in different tissues were measured. Under the Cd stress, the expression showed a trend of leaf > stem > root (Figure 7A). This indicates that the response of *StMTP*s in leaves are faster than that of roots and stems after being stressed by heavy metals in the flowering stage of potato. When potatoes were subjected to cadmium stress, StMTP3 in roots was significantly up-regulated at 6 h, which was 3.61 times that of the control (0 h). The expression level of StMTP1 reached the highest at 12 h, which was 2.55 times that of control. The other genes in the root did not show a significant up-regulation. In stem tissues, the expression level of StMTP11 was the highest at 6 h, reaching 13.59 times that of the control. However, StMTP2 and MTP8 have been down-regulated in the stem tissues no matter at any point in time. In leaf tissues, most *StMTP*s are up-regulated. The StMTP9 and StMTP10 were up-regulated extremely significantly, reaching around 24 times of the control.

Under Zn treatment, except for *StMTP1* and *StMTP3* in the stem tissues, the other genes were not significantly up-regulated regardless of the time period. The interesting thing is that *StMTP2* and *StMTP8* have been down-regulated in the stem tissues no matter at any point in time like. The response pattern of *StMTP8* and *StMTP2* to Zn stress in the stem is the same as that of Cd, and both exhibited a down-regulation phenomenon at any time. In the leaves, the expression level of *StMTP3* was the highest, reaching 5.23 times that of the control. In summary, *StMTP3* is more sensitive to Zn stress.

In general, under Cd^2+^ stress, the expression levels of *StMTP9*, *StMTP10*, and *StMTP11* were significantly up-regulated (Figure 7B). The expression levels of *StMTP3*, *StMTP9* and *StMTP11* were obviously higher than other genes under Zn^2+^ stress. In contrast, some genes represented by StMTP8 are not sensitive to Zn and Cd stress. The up-regulation of their expression may be specifically induced by metal ions, suggesting that they may play a role in the transport and absorption of heavy metals.

### 2.8. Yeast Complementation Assay

**Figure 8:**
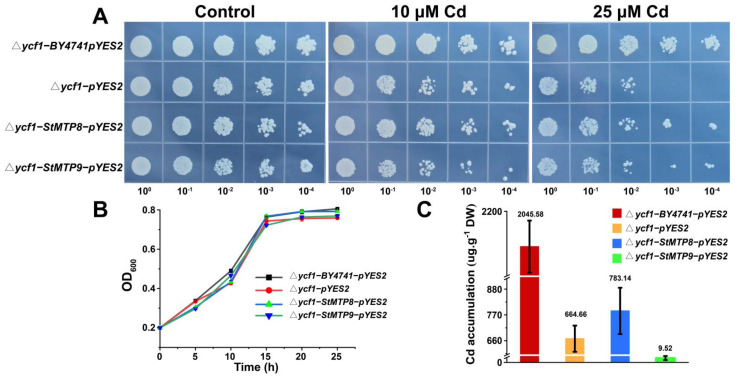
Functional analysis of *StMTP8* and *StMTP9* in the yeast cells. (**A**): *Δycf1* and BY4741 (control) carrying empty vectors and recombinant plasmids carrying *StMTP8* and *StMTP9* recombinant plasmids were grown on SG solid medium containing 0, 10, 25 μM Cd for 3 days. (**B**): The growth status of yeast cells at different time points. Culture the yeast OD_600_ to 0.2, add CdCl_2_ to make the medium concentration reach 25 μM, then take samples and measure every 5 h. (**C**): The accumulation of Cd in yeast cells. The different colored bars indicate different yeast strains, and the numbers on the bars represent the content of Cd in the yeast.

*StMTP9*, which actively responds to Cd stress, and *StMTP8*, which has a low expression level under Cd stress, were selected for ectopic expression in yeast cells. The spotting experiment results show that there is no significant difference in the growth of yeast cells without Cd and 10 μM Cd treatment. When 25 μM Cd was provided to the culture medium, StMTP8, StMTP9 (Figure 8A) improved the sensitivity of *∆ycf1* to Cd. The growth curve results showed that the growth rate of yeast carrying *StMTP*s were slightly better than *∆ycf1*-pYES2. Interestingly, *StMTP8* contains more cadmium than *∆ycf1* after being treated with 25 μM Cd for 48 h, but the cadmium in StMTP9 is significantly lower than *∆ycf1* (Figure 8C). These results show that the expression of *StMTP8* and *StMTP9* in yeast enhances the tolerance of yeast cells to Cd, and *StMTP9* may be involved in the efflux of Cd to the external medium for detoxification.

## 3. Discussion

### 3.1. Evolutionary Analysis of Potato MTP Family

The potato MTP family has fewer members, and collinearity analysis shows that there is no large-scale gene doubling. There is only one pair of collinear gene pairs between potato and model plants rice and Arabidopsis, indicating that *StMTP*s have undergone specific differentiation before the evolution of monocotyledonous and dicotyledonous plants, which means that their functions are specialized [35]. In particular, *StMTP5* still has collinearity gene pairs in model plants Arabidopsis and rice (such as: *StMTP5*: *AtMTP11*; *StMTP5*: *OsMTP11*), which represents the functional conservation of *StMTP5* and retains the functions of the original ancestor. The function of *StMTP5* may be closely related to the reported *AtMTP11* and *OsMTP11* [36]. For example, *AtMTP11* can increase the tolerance of yeast to heavy metals [37]. *OsMTP11* can chelate heavy metals into the Golgi apparatus and excrete them from the cell through exocytosis [38]. *StMTP5* may also have the function of transporting heavy metals to the Golgi and excreting them outside the cell, which can be further studied as an important candidate gene. According to the gene structure and motif characteristics of *StMTP*s, combined with the classification of model plants (Arabidopsis, rice) [39]. The members of the potato MTP family were divided into three subfamilies and seven groups. Surprisingly, *StMTP*s are lost in the Zn/Fe-MTP subfamily and are only evenly distributed in the Zn-MTP and MN-MTP subfamily. It indicates that the Zn/Fe-MTP subfamily may be lost during the potato gene evolution process without being activated by the natural environment or frameshift mutations for a long time. The phenomenon is worthy of further exploration by geneticists. The phylogenetic tree showed that *StMTP*s *2/4* and *AtMTPs 12/5* may have similar functions. According to reports, *AtMTPs 5/12*, as well as the *MTP5* and *MTP12* in cucumber, can form functional heterodimers, transport Zn^2+^ to the Golgi apparatus and participate in the outflow of Zn from the cytoplasm of yeast cells [40,41]. We speculate that StMTPs 2/4 can dimerize and transport heavy metals to the outside of the cell. In summary, the members of the *StMTP* family have conserved but diverse functions and play a vital role in the evolution of functions in response to environmental stress.

### 3.2. Conservative StMTP9 and Specific StMTP8

*StMTP8* is distributed in G1 whose members are related to the transport of Zn [42]. However, the results of qRT-PCR did not find an increase in the expression of *StMTP8* under zinc stress. The *StMTP9* exists in G9 whose members mainly respond positively to Mn^2+^ stress [43]. Interestingly, the expression of this gene increased significantly under Cd stress. The reason for these phenomena may be related to the structure of these two genes. According to the alignment of the StMTP protein sequences, the Mn-MTP subfamily has a core sequence, which is the highly conserved DXXXD. Highly conserved HXXXD residues exist in the Zn-MTP subfamily. It is reported that the two conserved residues are mainly distributed on TMD2 or/and TMD5, which can participate in metal transport [44,45]. There is a DXXXD domain on TMD2 of StMTP9, while the HXXXD of StMTP8 is lost in TMD2 or/and TMD5, which means that the transport function of StMTP8 to metal ions may have changed. The zinc-rich region can also be found in the protein sequence of StMTP8, which plays a key role in the selection and differentiation of metal ions [46,47]. In addition, we found a dimer domain (Motif2) related to Zn transporters in *StMTP9* [48]. Interestingly, a signal peptide was found on the first motif of *StMTP8*. The signal peptides are short amino acid sequences at the amino terminus of proteins that target proteins into or across membranes [49]. In general, *StMTP9* is representative, and *StMTP8* has specific mutations.

### 3.3. Co-Expression Network Analysis under Cd and Zn Stress

It is reported that Cd^2+^ is easily absorbed by plant roots through Zn^2+^ transporter, so it is speculated that these genes are related to Cd stress [50,51]. Therefore, it is speculated that the gene that can transport Zn is also related to Cd stress. This speculation has also been confirmed. For example, the *OsHMA2* is the main transporter of Zn and Cd from root to shoot [52]. The expression of *MTP1.3*, *MTP2* and *MTP7.1* in radish can be up-regulated under Zn and Cd stresses [53]. The expression of *OsMTP1* in yeast confers tolerance to Zn and Cd [54,55]. In this study, we found that *StMTPs 3/9/10/11* can respond to both Zn and Cd stresses. On the contrary, *StMTP8* is not sensitive to Zn and Cd stresses. Through comparison, we found that the genes that respond to both Zn and Cd stress are located in Mn-MTP, and their structures are relatively conservative. The conserved DXXXD residues are also found on TMD2. Among them, StMTP9 contains all the motifs of the remaining three members. But the structure of StMTP8 has undergone major changes (Table 2). These changes may cause it to fail to respond to Zn or Cd stress in a timely manner. Based on the above, we chose *StMTP*s *8/9* to preliminarily verify their functions in yeast.

### 3.4. Special Cadmium Tolerance of StMTP9 in Yeast Mutants

At present, yeast mutants are widely used to identify the basic functions of genes under heavy metal stress [56,57]. *StMTP9* can significantly respond to Cd^2+^ stress, but the expression of *StMTP8* does not change significantly under Zn or Cd stress. To our surprise, through heterologous expression in yeast, we found that both the expression of *StMTP9* and *StMTP8* can increase the tolerance of *∆ycf1* to Cd. What makes us happy is that *StMTP9* reduces the Cd content in cells, which may be related to the conserved amino acid residues DXXXD and motif2. Because the highly conserved aspartyl residue on TMD2 is the selective binding site of Zn^2+^/Cd^2+^ [58,59]. There is a ZT_dimer in motif2, which is reported to be the dimerization region of the entire zinc transporter molecule. It is speculated that *StMTP9* can form homodimers or heterodimers to transport Cd^2+^ out of the cell [60]. These two structures were not found in *StMTP8*, but a signal peptide was found on its first motif. Its appearance may affect the function of *StMTP8*.

### 3.5. Conclusions

This study identified 11 *StMTP*s from the potato genome for the first time. The systematic omics, comparative omics and other methods were used to analyze them, and five relatively conservative or special genes were summarized. In particular, we found that *StMTP9* has a relatively conservative structure, and *StMTP8* has undergone specific structural changes. Yeast heterologous expression found that these two genes both increased the tolerance of *∆ycf1* to Cd. To our surprise, the expression of *StMTP9* can reduce the cadmium content in yeast, while *StMTP8* does the opposite. This study discovered the gene *StMTP9* that can be used as a candidate for low Cd accumulation, which provides an important genetic resource for potato safety production and stress resistance research. However, the key residues that StMTP9 binds to Cd and the molecular mechanism of how Cd is transported from the cell to the outside of the cell need to be further validated and studied.

## 4. Materials and Methods

### 4.1. Identification of MTP Gene Family Members and Collinearity Analysis

The 2019 ensemble database (https://sep2019-plants.ensembl.org/index.html, accessed on 13 September 2021) was used to download the genome and protein files of *Solanum tuberosum*, *Solanum* *lycopersicum*, *Arabidopsis thaliana*. The pfam database (http://pfam.xfam.org/family/PF01545, accessed on 13 September 2021) is the source of potato Hidden Markov Model (HMM). The MTP domain candidate sequences were obtained by the HMM search program of BioLinux system HMMER (v3.1). The Evalue is set to 1.2e-10. We used the SMART (http://smart.embl.de/, accessed on 13 September 2021), NCBI CDD (https://www.ncbi.nlm.nih.gov/cdd/, accessed on 13 September 2021) and pfam (http://pfam.xfam.org/, accessed on 13 September 2021) confirmed candidate MTPs in potato, tomato and Arabidopsis. The identified MTP family protein sequences in rice were downloaded from phytozome (https://phytozome.jgi.doe.gov/pz/portal.html, accessed on 13 September 2021). After processing the location information of genes, the online software Map Gene2Chromosome v2 (http://mg2c.iask.in/mg2c_v2.0/, accessed on 13 September 2021) was used to draw the chromosome location map. The homology between potato, tomato, Arabidopsis and rice MTP genomes was analyzed using multiple collinearity scan (MCScan) [61].

### 4.2. Phylogenetic Analysis of the MTP Gene Family

Compared the protein sequences in MEGA-X (v10.1.8). Then, the phylogenetic trees of potato, tomato, Arabidopsis and rice family members were constructed by the Neighbor Join method (N-J). The bootstrap value which was used to evaluate the reliability of internal branches is set to 1000. The online software Evolview (https://doi.org/evolgenius.info//evolview-v2/#login, accessed on 13 September 2021) was used to visualize the evolutionary tree data.

### 4.3. The Gene Structures and Motif Analysis in Potato and Arabidopsis

The bio-Linux system was used to obtain the location information of Introns-exons and UTR of MTP genes in potato and Arabidopsis. The Tbtools (v1.0692) is used for drawing. The meme software analyzes the motif analysis of potato and Arabidopsis protein sequences, and then imports the analysis results into TBtools (v1.0692) for mapping. We have identified the domains included in Motif in the pfam database (http://pfam.xfam.org, accessed on 13 September 2021). Adobe Illustrator CC 2019 software was used to combine the gene structure map with the conservative motif map.

### 4.4. The Characterization of Predicted StMTP Proteins

The online analysis tool ExPASyProtParam (https://web.expasy.org/protparam/, accessed on 13 September 2021) was used to analyze the physical and chemical properties of StMTPs [62]. Plant-mPLoc (http://www.csbio.sjtu.edu.cn/bioinf/plant-multi/, accessed on 13 September 2021) was used to predict the subcellular locations of proteins and the TMHMM Server v. 2.0 was used to predict the domains of the StMTP family members.

### 4.5. Comparison of Potato and Arabidopsis Protein Sequences

clustalx-2.0.11 compared the proteins of potato and Arabidopsis. GeneDoc software is reserved for further analysis of the comparison results. The representative genes among G5, G12, G1, G6, G7, G9, and G8 were selected to continue in-depth discussion. They were imported into Protter (http://wlab.ethz.ch/protter/start/, accessed on 13 September 2021) for analysis. The motif was marked with different colors on the protein sequence.

### 4.6. Analysis of Cis-Acting Elements of StMTPs

After the 2000 bp sequence upstream of the transcription start site of *StMTP*s family members was extracted, and then the Plant CARE (http://bioinformatics.psb.ugent.be/webtools/plantcare/html/, accessed on 13 September 2021) were used to predict the promoter *cis*-acting elements [63]. The heat map in TBtools was used to produce images of the gene promoters.

### 4.7. Obtaining and Processing Methods of Test Materialss

This experiment used Yunshu 505 (*Solanum tuberosum*) as the material. Potted plants in the experimental base of Guizhou University in June 2020, then using about 100 mg/kg CdCl_2_, 100 mg/kg ZnCl_2_ to stress potato for 0, 6, 12 and 24 h [64]. After the middle leaves, roots and stems of the potato plants are harvested, they are placed in liquid nitrogen and stored in a refrigerator at −80 °C. The Total RNA Extraction Reagent (Vazyme Biotechnology Co., Ltd., Nanjing, China) was used to isolate the total RNA from roots, stems and leaves. The StarScript II First-strand cDNA Synthesis Mix with a Gdna Remover kit (GeneStar, Beijing Kangruncheng Biotechnology Co., Ltd., Beijing, China) was used to synthesize cDNA. The CFX96 Real-time PCR System (CFX96, BIO-RAD, California, USA) measured the expression of *StMTP*s. The internal reference and target gene sequences were simultaneously amplified using the cDNA as a template. Each of them was repeated three times. A 20 μL reaction mixture was used in this experiment, which contained: 10 μL of 2 × RealStar Green Fast Mixture (GeneStar, Beijing Kangruncheng Biotechnology Co., Ltd., Beijing, China), 1 μL of primer, 7 μL of RNase-Free ddH_2_O and 2 μL of cDNA. A two-step protocol was used that consisted of 95 °C for 3 min, 95 °C for 15 s and 65 °C for 15 s for a total of 40 reaction cycles. Data analysis was calculated using 2^−ΔΔCt^. Origin 2018 64Bit and Cytoscape (v3.6.1) was used to establish and analyze the influence of heavy metals on *StMTP*s expression.

### 4.8. Yeast Complementation Assay

Wild-type yeast BY4741 (*MATα*; *his3Δ1*; *leu2Δ0*; *met15Δ0*; *ura3Δ0*) and its mutant *∆ycf1 (MATα*; *his3Δ1*; *leu2Δ0*; *met15Δ0*; *ura3Δ0*; *YDR135c: kanMX4*) were obtained from the Euroscarf (http://www.euroscarf.de/shoppingCart.php#, accessed on 13 September 2021). They were used for ectopic expression of *StMTP8* and *StMTP9*. The two *StMTP*s were amplified and ligated into the yeast vector by homologous recombination. The recombinant plasmid and empty vector (control) were transformed into yeast strain cells. The transformed yeast is cultured in a glucose (SD-U) liquid nutrient medium lacking uracil [65,66]. When the OD_600_ (600 nm optical density) is 0.7–0.8, serial dilution (OD_600_ = 10^0^, 10^−1^, 10^−2^, 10^−3^ and 10^−4^). The diluted yeast cells were placed on a galactose solid medium (SG-U) lacking uracil and containing 0, 10, 25 μM Cd [67], incubated inverted at 30 °C for 3 days, and then photographed and recorded. Yeast was cultured in SG-Ura liquid medium (20 mL) to an OD_600_ of 0.2, treated with 25 μM Cd, and OD_600_ was measured every 5 h to determine the growth curve of the transformant. In order to determine the cadmium content in yeast cells, they Were cultured in SG-U (100 mL) to OD_600_ = 0.2, then treated with 25 μM Cd and cultured for 48 h. The cells were collected and washed three times with distilled water. Then they were dried at 85 °C for 2 days and digested with 5 mL of nitric acid. The metal concentration is measured by ICP-OES ((ICP-OES-7000, Thermo Fisher Scientific, New York, USA).

## Figures and Tables

**Table 1:** The basic physical and chemical properties of *StMTP* family in potato.

Gene Name	Transcript ID	MW (KDa)	PI	Instability Index	GRAVY	Subcellular Localization	TMD Number
*StMTP1*	PGSC0003DMT400081988	45.43	5.07	43.26	0.0300	Vacuole	5
*StMTP2*	PGSC0003DMT400017877	100.11	6.97	42.20	−0.0400	Vacuole	16
*StMTP3*	PGSC0003DMT400002845	44.83	5.01	48.32	0.1200	Vacuole	4
*StMTP4*	PGSC0003DMT400038744	28.77	7.17	46.81	−0.2100	Vacuole	2
*StMTP5*	PGSC0003DMT400060459	38.96	5.22	44.16	0.2400	Vacuole	4
*StMTP6*	PGSC0003DMT400077991	42.50	5.85	28.80	0.1400	Vacuole	6
*StMTP7*	PGSC0003DMT400078896	45.97	6.05	28.74	0.0050	Vacuole	6
*StMTP8*	PGSC0003DMT400078996	39.47	5.97	34.53	0.1900	Vacuole	5
*StMTP9*	PGSC0003DMT400029243	51.40	6.63	46.16	0.0300	Cell membrane; Vacuole	7
*StMTP10*	PGSC0003DMT400024996	40.00	8.96	42.90	−0.0200	Cell membrane; Vacuole	4
*StMTP11*	PGSC0003DMT400010945	46.38	5.35	46.56	0.0300	Vacuole	4

**Table 2:** Summary of the functional StMTPs.

StMTPs	Similar Functions to Model Plants	Structure Domain (HXXXD or DXXXXD)	Zinc-Rich Area	ZT_Dimer	Signal Peptide	Improve Yeast Cadmium Tolerance
StMTP2	Forms dimers with StMTP4 to transport Zn^2+^	HXXXD not exists on TMD2 or/and TMD5	Yes	No	No	-
StMTP4	Forms dimers with StMTP2 to transport Zn^2+^	HXXXD not exists on TMD2 or/and TMD5	No	No	No	-
StMTP5	Transport the heavy metals	DXXXD exists on TMD2 and TMD5	No	Yes	No	-
StMTP8	Transport Zn^2+^	HXXXD not exists on TMD2 or/and TMD5	Yes	No	Yes	High tolerance; high accumulation
StMTP9	Transport Mn^2+^	DXXXD exists on TMD2	No	Yes	No	High tolerance; low accumulation

## Data Availability

Not applicable.

## Supplementary Materials

The following are available online at https://www.mdpi.com/article/10.3390/ijms221910478/s1.
